# Improved Detection of Rare Genetic Variants for Diseases

**DOI:** 10.1371/journal.pone.0013857

**Published:** 2010-11-08

**Authors:** Lei Zhang, Yu-Fang Pei, Jian Li, Christopher J. Papasian, Hong-Wen Deng

**Affiliations:** 1 Center of System Biomedical Sciences, University of Shanghai for Science and Technology, Shanghai, People's Republic of China; 2 Key Laboratory of Biomedical Information Engineering, School of Life Science and Technology, Ministry of Education and Institute of Molecular Genetics, Xi'an Jiaotong University, Xi'an, Shaanxi, People's Republic of China; 3 School of Medicine, University of Missouri-Kansas City, Kansas City, Missouri, United States of America; 4 College of Life Sciences and Engineering, Beijing Jiao Tong University, Beijing, People's Republic of China; VU University, Netherlands

## Abstract

Technology advances have promoted gene-based sequencing studies with the aim of identifying rare mutations responsible for complex diseases. A complication in these types of association studies is that the vast majority of non-synonymous mutations are believed to be neutral to phenotypes. It is thus critical to distinguish potential causative variants from neutral variation before performing association tests. In this study, we used existing predicting algorithms to predict functional amino acid substitutions, and incorporated that information into association tests. Using simulations, we comprehensively studied the effects of several influential factors, including the sensitivity and specificity of functional variant predictions, number of variants, and proportion of causative variants, on the performance of association tests. Our results showed that incorporating information regarding functional variants obtained from existing prediction algorithms improves statistical power under certain conditions, particularly when the proportion of causative variants is moderate. The application of the proposed tests to a real sequencing study confirms our conclusions. Our work may help investigators who are planning to pursue gene-based sequencing studies.

## Introduction

Genotyping based genetic association analyses, which are dependent on indirect linkage disequilibrium (LD) mapping, rely on the common disease-common variants (CDCV) hypothesis. The CDCV hypothesis assumes that genetic variants responsible for complex diseases are common in frequency in the human population [1], [2], [3]. Despite successful utilization of genome-wide association studies (GWAS) to identify susceptible common variants for a variety of complex diseases [4], only a modest fraction of phenotypic variation has been accounted for by the identified variants. Therefore, it is reasonable to propose that there are limitations to the CDCV hypothesis, and to consider the alternative common disease-rare variants (CDRV) hypothesis as a complement [5], [6], [7], [8]. In the CDRV hypothesis, phenotypic variation is attributable to multiple variants, each with a low frequency and a small to moderate marginal effect.

Advances in next-generation sequencing technologies [9], [10], [11], [12] and the recently launched ‘1000 Genomes Project’ [13] have enhanced our ability to discover rare variants. While knowledge regarding rare variants is becoming increasingly detailed, statistical analyses of rare variants present a number of challenges. Ordinary variant-by-variant methods that are suited for analyses of common variants have a limited capacity to detect rare variants due to their extremely low frequency. Additionally, the high number of tests to be examined reduces statistical power dramatically when correction for multiple testing is taken into account. To circumvent these problems, the analytical strategy of grouping variants has been suggested [14], [15], [16], [17]. Grouping strategy uses genomic regions (e.g. genes, that contain a set of variants) as the unit of analysis. This strategy is advantageous because it enhances the mutation signal as well as reduces the number of tests. Nonetheless, as not all nonsynonymous rare variants within genes are causative, grouping all of them regardless of their functional impact may have the undesirable effect of producing an unsatisfactory signal-to-noise ratio. Therefore, successful use of the grouping strategy is critically dependent upon the ability to distinguish potential causative variants from background population variation prior to analysis.

The potential functional importance of non-synonymous variants, or of the resulting amino acid substitutions, can be studied from a biological perspective. The functional impact of amino acid substitutions can be predicted by specialized algorithms that take into account context information for the specific protein being analyzed [18], [19], [20], [21], [22], [23], [24], [25], [26], [27]. By incorporating information obtained from predicting algorithms into association analyses, potentially causative variants can be preliminarily distinguished from background population variation, thereby improving the efficiency of these analyses.

In this study, we test the association between non-synonymous variants in gene coding regions and disease. In order to determine the relative importance of variants in terms of phenotypic effect, prediction algorithms were extensively utilized to identify functional amino acid substitutions, and we incorporated results from these prediction algorithms into association tests. We aimed to evaluate whether, and to what extent, incorporation of results from these prediction algorithms can improve statistical power for detecting disease associated rare variants. We also evaluated the performance of tests by analyzing a real sequence dataset to demonstrate the utility of the proposed tests.

## Methods

**Figure 1:**
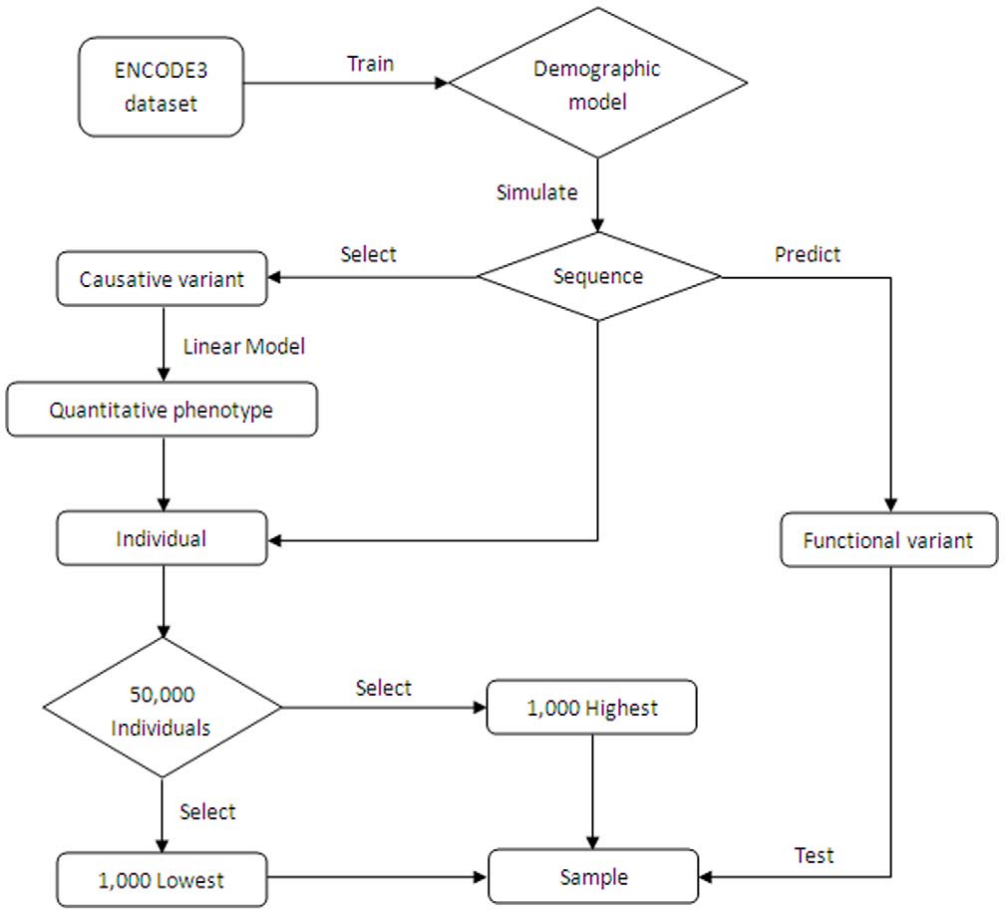
Flow chart of simulation studies.

In figure 1, we outline the steps that we follow to simulate data and perform association test. In what is followed at this section, we will describe each of these steps in details.

### Modeling site-frequency spectrum

By focusing on the European population, we simulate the full range frequencies of sequence data using a four-parameter demographic model [14], [28]. In this model, the shape of the European population history is assumed to start from a constant ancestral population, followed by a population bottleneck with a reduction in effective size, and then by an exponential expansion until to modern population. The four parameters involved are the constant ancestral population size *N*_1_, the bottleneck population size *N_b_*, the duration of time *T* after the bottleneck (measured by generation), and the population growth rate 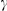 after the bottleneck. In Appendix S1, we outline the formulation of the demographic model and the data we use to estimate its parameters.

### Simulating genotypes

Using the inferred demographic model, we simulate the full range allele frequencies of sequence data of a gene coding region to mimic a gene-wise sequencing study. We simulate a gene with *L* = 1,500 nucleotides by assuming an average length of 500 amino acids for proteins [14]. In accordance with a previous estimation [29], one third of the simulated variants are assumed to be synonymous mutations, which does not affect gene expression and are not included in our analysis. Of the remaining non-synonymous mutations, previous studies have indicated that the majority are actually background population variation [30], [31]. We thus set the proportion of causative variants *f* = 0.2 [31]. However, we also evaluate two other proportions (0.5 and 0.8) for comparison.

### Simulating the pool of quantitatively phenotyped individuals

Though we test association with a case/control study design, individuals are sampled from the two tails of the distribution of a quantitatively phenotyped population. This extreme sampling scheme is advantageous because enlarging the size of phenotyped pool alone could improve statistical power to detect the association; this approach has been widely adopted by several well-established sequencing-based association studies [14], [32], [33], [34]. As a baseline, we generate a pool with 50,000 quantitatively phenotyped individuals. Causative variants are randomly selected, and they affect the phenotype in a cumulative matter in that individuals carrying more causative mutations have larger shifts of the phenotypic mean. Specifically, for each individual *j*, define a causative score 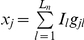, where *L_n_* is the number of non-synonymous rare variants; *I_l_* (1 or 0) is the indicator of causative variant, and *g_jl_* (0, 1, or 2) is the genotype score at the variant. The relationship between the phenotype and genotype is modeled by a linear regression equation
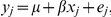
where *μ* is the grand phenotypic mean; *e_j_* is the random error which follows a normal distribution with zero mean. The coefficient *β* is determined by the locus heritability *h* ^2^, which is defined as
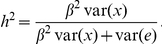
As a baseline, we set *h* ^2^ to be equal to 0.5%.

When common variants are selected as causative, they affect the phenotype individually by the above linear regression equation. Of the total 0.5% locus heritability, 10% is attributable to common variants.

### Sampling extreme phenotyped individuals for sequencing

After the pool of phenotyped individuals is generated, we select individuals with lowest and highest phenotypes for sequencing. As a baseline, we select *n_u_* = 1,000 individuals with the lowest phenotypes and label them as control subjects. Similarly, we select *n_c_* = 1,000 individuals with the highest phenotypes and label them as case subjects.

### Predicting the functional impact of non-synonymous SNPs

Since not all non-synonymous mutations are causative, we add a step to differentiate functional variants from background neutral variation based on the specific amino acid substitutions using existing prediction algorithms, e.g., PolyPhen [19] and SIFT [21]. We assume that functional variants are all causative. The information obtained from predicting algorithms can be used to generate qualitative (e.g., the variant results in damaged protein) or quantitative (e.g., a continuous score reflecting the degree of impact on protein function) measures. We assume that both measures are available. For simplicity, we take an indicator *O* as the qualitative measure of being functional (*O* = 1) or not (*O* = 0), and we take a probability *p* (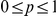) as the quantitative measure, though the bounds 0 and 1 on the range of measure are not required.

The performance of a specific predicting algorithm is measured by its sensitivity, e.g., the probability of correctly identifying a truly functional mutation, and specificity, e.g., the probability of incorrectly classifying a non-functional mutation as a functional one. Assume that the sensitivity and the specificity are 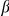 and 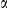. Specifically, we have 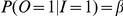, and 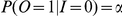. Thus, *O* is simulated from a Bernoulli distribution 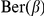 for causative variants, and from 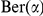 for neutral variants. Given *O* = 1 or 0, we simulate quantitative measure *p* from the Uniform distribution U(*a*, 1) or U(0, *a*), where *a* is the threshold to declare a causative variant. We set *a* = 0.9.

Estimations of sensitivity and specificity are available for most predicting algorithms [27]. The sensitivity ranges from 0.69 to 0.88, and specificity from 0.08 to 0.2. In this study, we set them to average values, e.g., sensitivity to 0.8 and specificity to 0.15, unless otherwise specified.

### Testing association

Individual rare variants, e.g., MAF<1%, provide limited information to detect the association, and an effective approach to analyze them is to group them together [15], [16]. The first step for analyzing a group of rare variants involves encoding a genotypic score, *s_j_*, for each individual *j* in the group. An infinite number of encoding schemes are available. Here we study three encoding schemes. In the first one, the genotypic score is the sum of individual genotypes
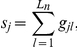
where *L_n_* is the number of rare non-synonymous variants, and 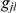 (0, 1, or 2) is the genotype at the *l*th variant for the *j*th individual. This encoding scheme is also adopted by a recent study [35]. The second and third schemes involve weighting each variant by its score simulated in the previous section. Denote, for each variant *l*, *p_l_* and *o_l_* as the continuous and binary functional measures. The genotypic score is given by
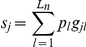
for the second encoding scheme, and is given by
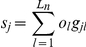
for the third scheme.

The association between the phenotype and encoded genotypic score is then examined by a logistic regression model. Specifically,
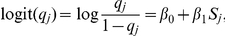
where *q_j_* is the risk of developing the disease for the individual.

Common variants can be analyzed in the combination with grouped rare variants in the above logistic regression model. Denote 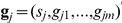 as the vector of the combination of rare and common variants, where *m* is the number common variants. The above logistic regression test is rewritten as
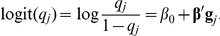



We denote tests with the three different encoding schemes as sum test (ST), functional variant weighted continuous sum test (FWCST) and functional variant weighted binary sum test (FWBST).

For comparison, we include two extensive tests that involve grouping rare variants for testing association. The first is the collapsing and its relevant combined multivariate collapsing (CMC) tests [15]. In the collapsing method, genotypic score is an indicator of the presence of rare variants
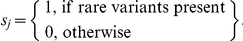
The association between the disease status and genotypic score is examined by the Fisher's exact test or 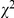 test. When common variants are involved, CMC was proposed to test the combination of common and grouped rare variants in a multivariate model, such as Hotelling's *T* ^2^ test [15].

The second test is the group-wise weighted sum statistic (GWWS) [16]. In GWWS, the genotypic score is another weighted sum of individual genotypes
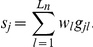


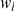
 is assigned so that rarer variants get heavier weights, because rarer variants tend to have a larger effect on phenotype. The association is examined by a rank-sum statistic, and the significance is evaluated by permuting and replicating the test *k*, e.g., 1000, times.

### Evaluating performance

We evaluate statistical properties, including type-I error rates and power, of various tests by simulation. Type-I error rates are estimated by setting the locus heritability to zero, and power is estimated by setting it to a specified proportion, e.g., 0.5%. Both type-I error rates and power are estimated on 10,000 replicates, where power is defined as the proportion of replicates in which the association is detected at the predefined significance level 1.0E-6. We also study the effects of several influential factors, e.g., the sensitivity and specificity of predicting algorithms, number of variants, and proportion of variants, on power estimates. When evaluating the effect of one particular factor, other parameters will be fixed at base line values.

### Application

As an application, we analyze the sequence data produced by Cohen et al. [34]. In their study, the authors sequenced three candidate genes (*ABCA1*, *APOA1*, and *LCAT*) in an initial sample with two groups of individuals to identify mutations that cause low high-density lipoprotein cholesterol (HDL-C) levels. The two groups each comprising 128 individuals were selected from the upper and lower 5% of the distribution of plasma HDL-C levels in the Dallas Heart Study (DHS) (Dallas sample). The authors identified a total number of 29 non-synonymous variants, but only 18 variants that were exclusive to either group were reported. As in [34], we combined variants of these three genes and analyzed them together. Two variants, each having four alleles in the 64 black individuals (Bayesian posterior frequency 3.8%), were considered as common variants, and the other 16 rare variants were grouped together.

The authors also sequenced the same three genes in a second sample comprising 155 white Canadians who had low levels of HDL-C and 108 who had high levels (Canada sample). A total number of 32 non-synonymous variants were identified, but only 23 variants, which were all rare, were reported and thus were available for us to analyze.

In their analysis, the authors predicted the impact of each variant on maintaining protein function using the predicting algorithm PolyPhen [19]. There are three qualitative measures available from PolyPhen: “benign”, “possibly damaging protein”, and “probably damaging protein”. We take “benign” variants as non-functional, and the other two types as functional. In addition, a position-specific independent counts (PSIC) profile score [36] from PolyPhen is available as a quantitative measure for each variant, which will be used in this study. Non-sense mutations, e.g., mutations that truncate proteins, could not be predicted by PolyPhen. As non-sense mutations probably change protein function, we category them as functional variants, and assign them the highest quantitative weight among others.

## Results

In this section, we first estimated the demographic model by analyzing the real sequence dataset produced by the ENOCDE3 project [37]. Based on the inferred demographic model, we then performed a series of simulation studies to evaluate the performance of several rare variant aimed association tests, including the proposed functional variant weighted continuous sum test (FWCST), binary sum test (FWBST), and sum test without weighting (ST). Two other existing tests, namely combined multivariate collapsing test (CMC) [15] and group-wise weighted sum statistic test (GWWS) [16], were also included in our analysis for comparison. We finally re-analyzed the real study reported by Cohen et al. [34].

### Estimating demographic model

**Figure 2:**
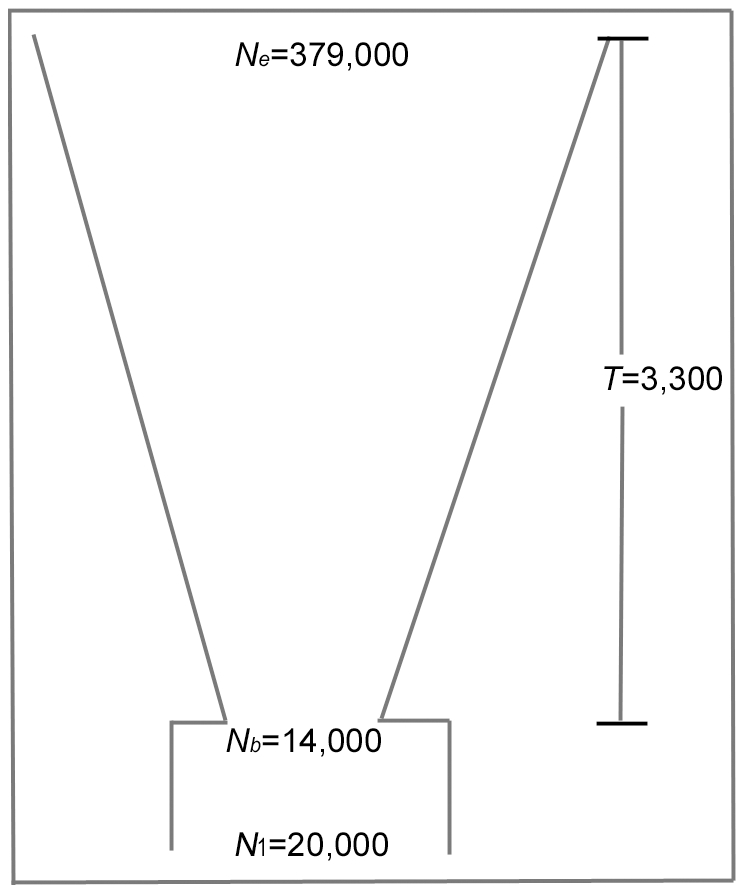
Demographic model. The constant ancestral population (*N*_1_) is followed by a population bottleneck with a reduction of effective size (*N_b_*), and then by an exponentially expansion with *T* generations, until to the present population (*N_e_*).

**Figure 3:**
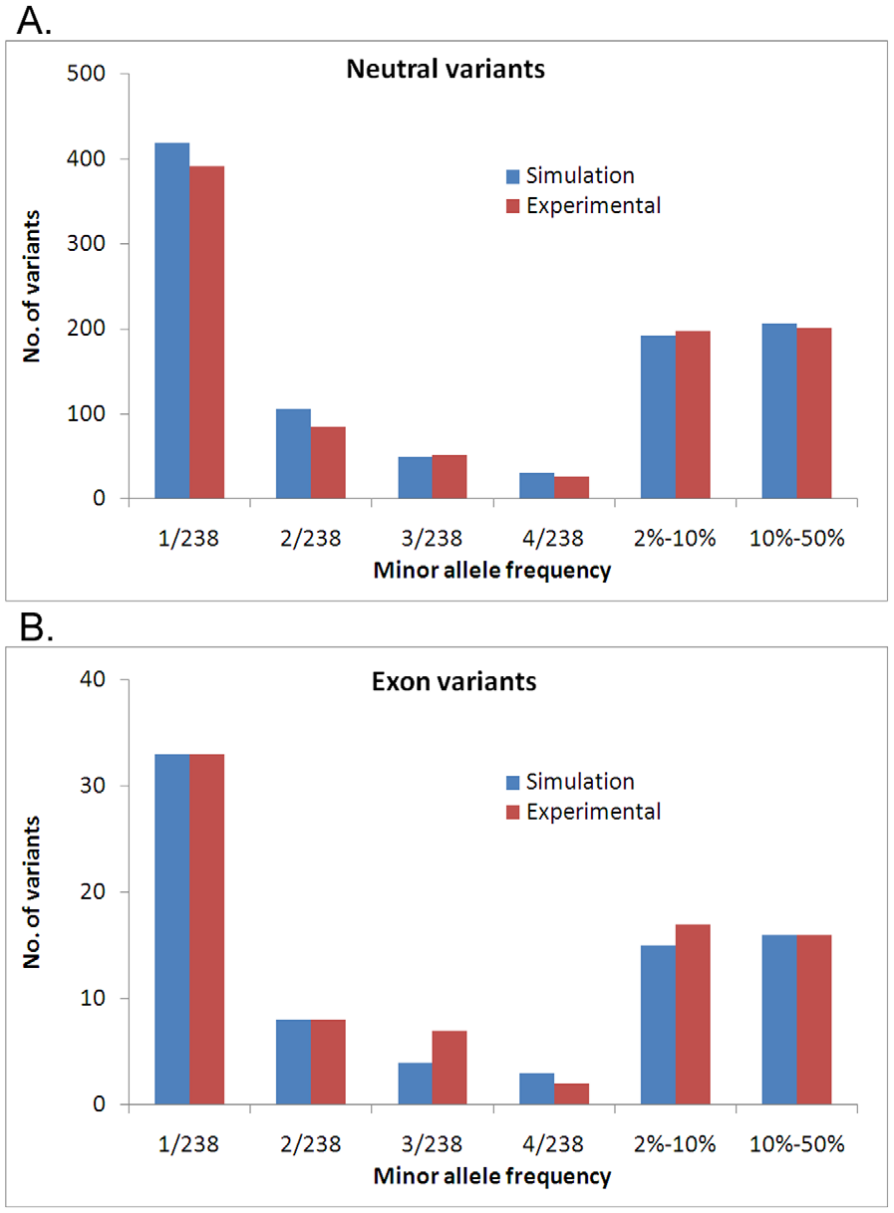
Fitness of simulated variants to experimental variants. The fitness of allele frequencies simulated by the demographic model to that of experimental data for neutral variants (*A*) and coding variants (*B*).

The four parameters of demographic model estimated by maximum likelihood estimation are *N*_1_ = 20,000, *N_b_* = 14,000, *T* = 3,300, and 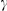 = 0.001 (See figure 2 for illustration). *N*_1_ and *N_b_* are larger than a previous report by two fold [38]. Given an average 20 years per human generation, the estimated *T* implies that the bottleneck occurred ∼66,000 years ago (66 kya). This estimation is consistent with the “out-of-Africa” event which presumes that the European branch was expanded from Africa ∼80–40 kya. The estimated effective size of today's population is ∼380,000, much larger than that reported in [38] (20,000), but smaller than that reported in [14] (900,000). For neutral variants the estimated allele frequencies from simulations matched quite well with experimental data (figure 3A). Due to the limited number of non-synonymous variants in this dataset, the strength of natural selection could not be evaluated. Instead, using a model with neutral selection, the frequency predicted by simulation for alleles in gene coding regions matched quite well with the experimental data from the ENCODE3 project (figure 3B). This may be because the effect of selection was too weak to be detected by the limited number of available non-synonymous variants.

### Testing association

#### Type-I error rates

As shown in table 1, all methods have reasonable type-I error rates that are close to target levels in all situations that were studied.

**Table 1:** Type I error rates.

Sequenced		Nominal level									
sample	Gene	5%					1%				
size	length	FWCST	FWBST	ST	GWWS	CMC	FWCST	FWBST	ST	GWWS	CMC
1,000											
	500	5.6	5.5	5.0	5.1	4.7	1.2	1.1	0.8	1.0	1.2
	1,000	5.1	5.5	4.9	5.1	5.2	1.2	1.1	1.1	1.0	1.1
	1,500	5.5	5.3	5.4	5.1	5.5	1.1	1.3	1.2	1.2	1.3
	2,000	4.8	5.1	4.8	4.9	4.5	0.9	0.7	1.2	0.9	1.0
	2,500	5.2	4.7	4.9	4.9	4.7	1.2	1.2	1.1	1.0	0.8
	3,000	4.7	5.4	4.9	4.8	5.5	1.1	1.2	0.8	0.9	1.3
2,000											
	500	5.5	5.7	5.1	5.0	5.3	1.0	1.1	0.9	1.0	0.8
	1,000	4.8	4.5	5.1	4.7	4.8	1.0	1.1	0.8	0.9	0.9
	1,500	4.2	5.0	4.5	4.5	5.0	0.8	0.9	0.9	1.0	1.0
	2,000	4.5	4.3	5.2	5.4	5.5	0.8	1.0	1.0	1.4	1.5
	2,500	4.8	4.3	5.3	5.5	5.1	0.8	0.8	1.2	1.1	1.2
	3,000	5.0	4.7	4.4	4.2	5.4	1.1	1.2	0.9	1.1	1.1
4,000											
	500	5.2	5.3	5.1	5.2	4.7	1.1	1.0	1.2	1.0	1.2
	1,000	5.5	5.2	5.0	4.8	4.9	1.1	1.1	0.9	0.8	1.0
	1,500	5.4	5.1	5.1	5.1	5.2	1.0	1.2	0.9	1.2	0.9
	2,000	4.6	4.8	4.5	4.8	4.5	0.8	1.0	0.8	0.8	1.0
	2,500	5.5	5.5	5.3	5.1	5.1	1.0	1.2	1.0	1.0	1.1
	3,000	4.9	5.0	4.4	4.5	4.5	0.8	0.7	0.8	0.8	0.8

Sequenced sample size varies from 1,000 to 4,000, while the gene length varies from 500 to 3,000 nucleotides. Type I error rate is estimated on 10,000 replicates at significance levels 5% and 1%.

Abbreviation: FWCST, the proposed functional variant weighted continuous sum test; FWBST, the proposed functional variant weighted binary sum test; ST, the sum test without weighting; GWWS, the group-wise weighted sum test [16]; CMC, the combined multivariate collapsing test [15].

#### Power with proportion of causative variants

**Figure 4:**
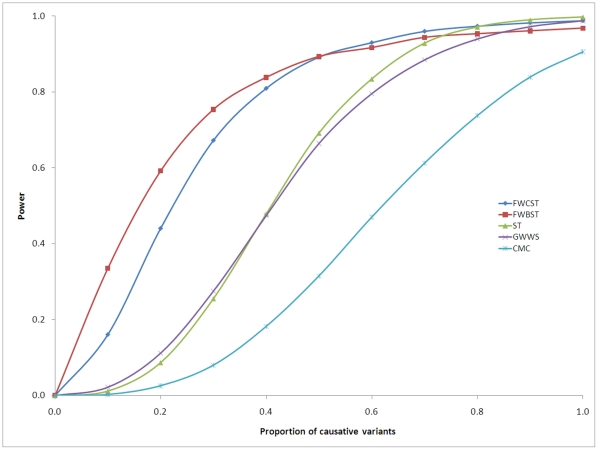
Power with proportion of causative variants. Genotype data of a gene with 1,500 nucleotides are simulated. A pool of 50,000 quantitatively phenotyped individuals is generated, from which 1,000/1,000 individuals with the lowest and highest phenotypes are selected and labeled as control/case subjects. Causative variants explain cumulatively 0.5% of the total phenotypic variation, of which 90% is explained by rare variants, and the remaining 10% by common variants. The sensitivity and specificity of prediction algorithms are set to 0.8 and 0.15, respectively. Power is estimated on 10,000 replicates, defined as the proportion of replicates in which the gene is detected at the significance level 1.0E-6. Abbreviation: FWCST, the proposed functional variant weighted continuous sum test; FWBST, the proposed functional variant weighted binary sum test; ST, the sum test without weighting; GWWS, the group-wise weighted sum test [16]; CMC, the combined multivariate collapsing test [15].

All methods had increased power as the proportion of causative variants increased; their relative performance depended on the proportion (figure 4). When the proportion was lower than 0.7, FWCST and FWBST had the highest power. When the proportion exceeded 0.7, however, the power of ST exceeded that of FWBST. When the proportion was as high as 0.9, the power of ST exceeded that of FWCST, and the power of GWWS exceeded that of FWBST. Comparing FWCST to FWBST, FWBST had higher power when the proportion was below 0.5 but, at levels above 0.5 FWCST had higher power. Comparing ST to GWWS, GWWS had higher power when the proportion was below 0.4 but, at levels above 0.4, ST had higher power. CMC had the lowest power under all tested conditions.

#### Power with predicting sensitivity

**Figure 5:**
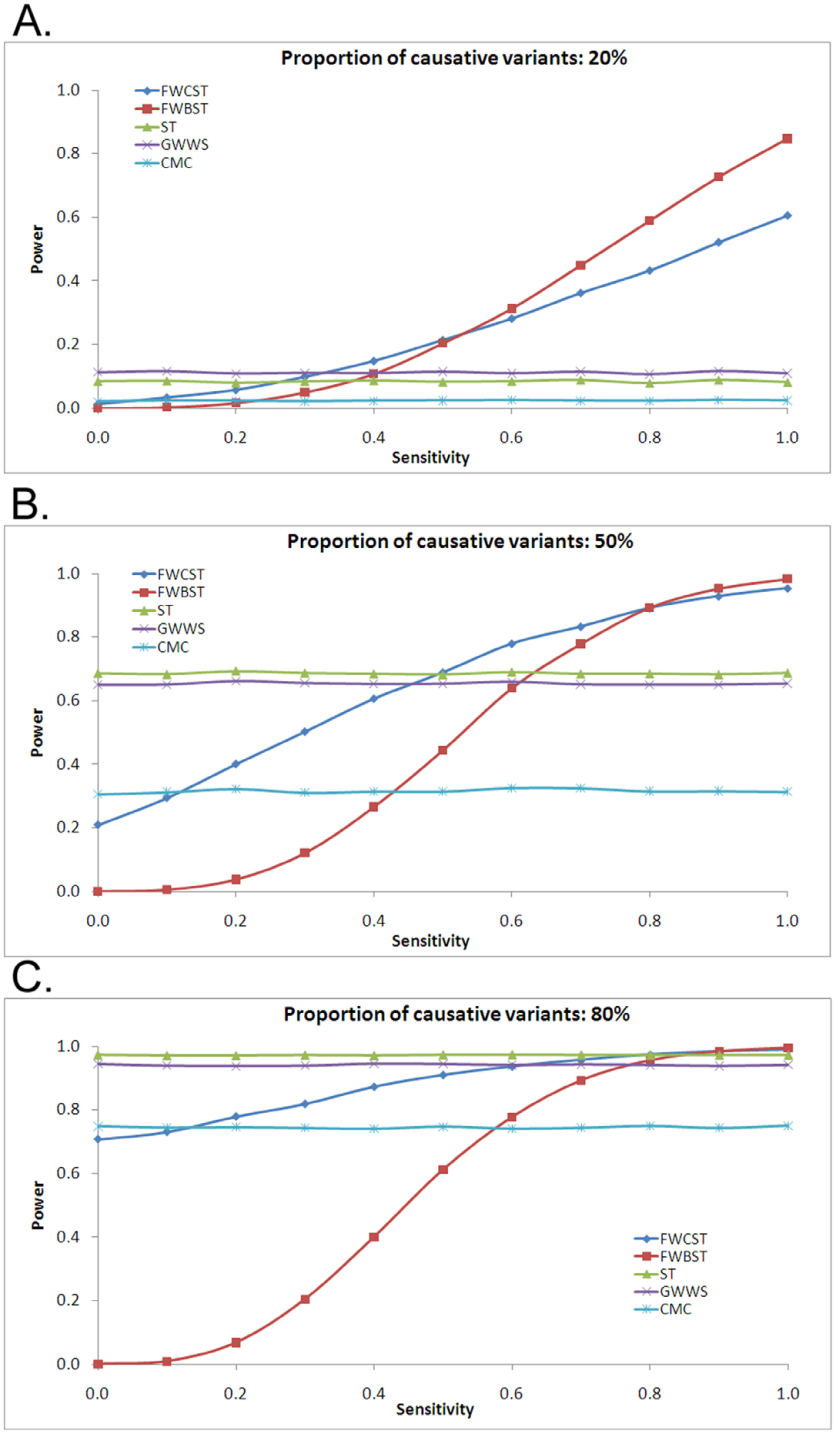
Power with sensitivity of predicting functional variants. Three proportions of causative variants are considered: 20% (A), 50% (B), and 80% (C). The specificity for identifying functional variants is set to 0.15. See the legend for figure 4 for abbreviations and simulation detail.

The sensitivity of functional variant prediction is defined as the probability of correctly identifying a truly functional mutation (Functional variants are assumed to be causative/relevant to phenotype). Sensitivity affected the performance of FWCST and FWBST in that power increased with greater sensitivity (figure 5). The relative performance of FWCST and FWBST to other tests also depended on the proportion of causative variants. At low (20%, figure 5A), medium (50%, figure 5B), and high (80%, figure 5C) proportions, their power exceeded that of other tests when sensitivity exceeded approximately 0.4, 0.6, and 0.8, respectively. When sensitivity was low, FWCST was more powerful than FWBST, but power increased more rapidly for FWBST than for FWCST with increasing sensitivity. Consequently, when sensitivity was high, FWBST was more powerful than FWCST. Among the other methods, GWWS and ST had approximately equal power, while the power of CMC was lowest.

#### Power with predicting specificity

**Figure 6:**
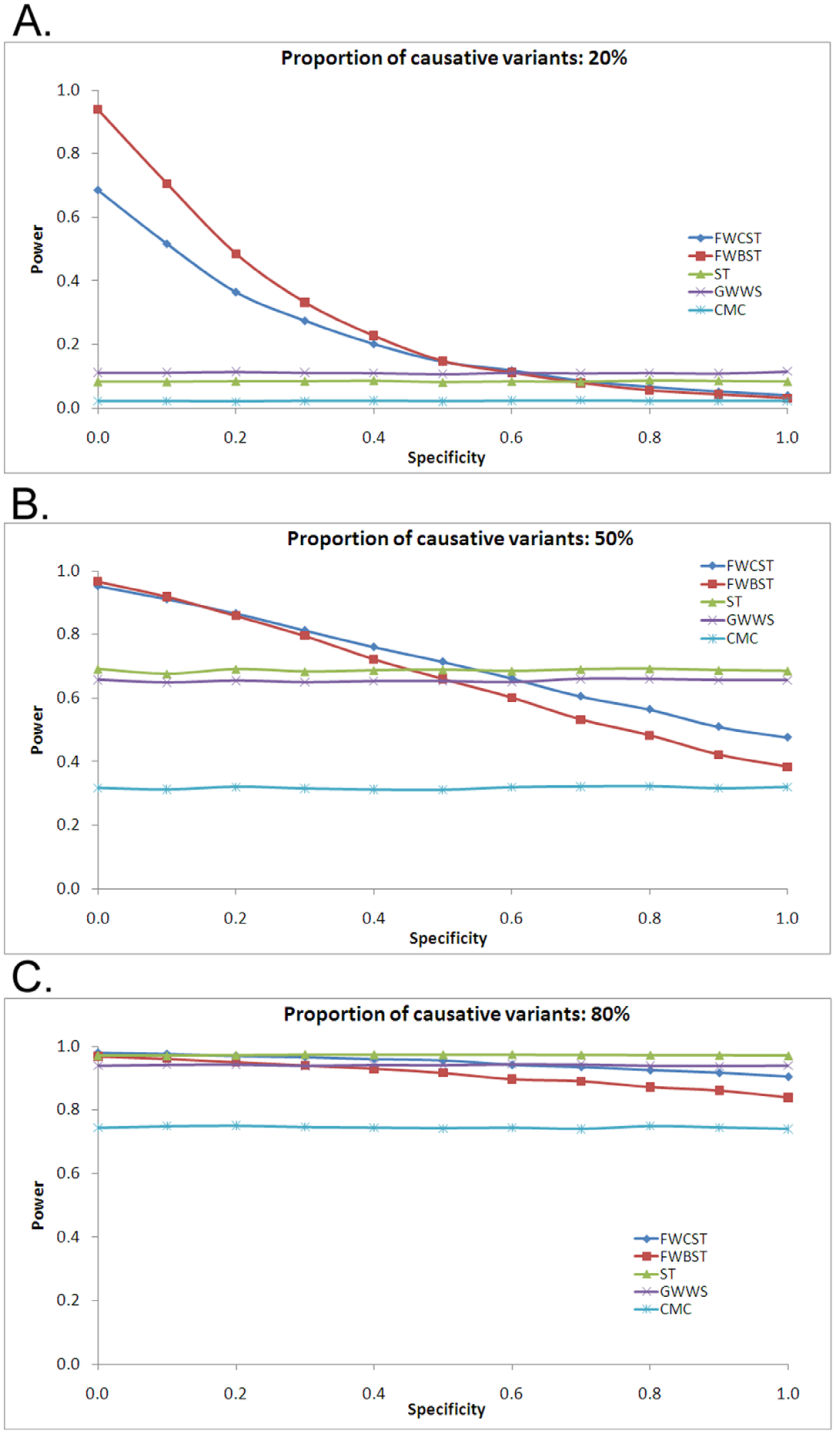
Power with specificity of predicting functional variants. The sensitivity for predicting functional variants is set to 0.8. See the legend for figure 4 for abbreviations and simulation detail.

The specificity of functional variant prediction is defined as the probability of incorrectly classifying a non-functional mutation as a functional one. Specificity affected the performance of FWCST and FWBST in that power decreased with increasing specificity (figure 6). Again, the relative performance among tests depended on the proportion of causative variants. At low proportion (figure 6A), the power of FWCST and FWBST was higher when specificity was lower than ∼0.6. At medium and high proportions (figure 6B&C), the specificity criteria for power improvement was around 0.5 and 0.3, respectively. GWWS and ST had approximately equal power, while that of CMC was lowest.

#### Power with effective proportion of causative variants

**Figure 7:**
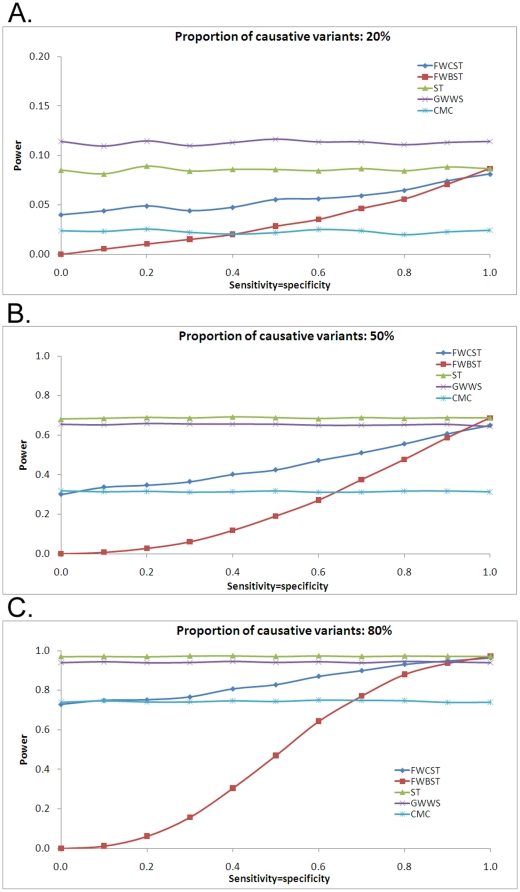
Power with equaling effective proportion of causative variants. The sensitivity and specificity are set to be equal, in order to retain the effective proportion of causative variants after weighting to that without weighting. See the legend for figure 4 for abbreviations and simulation detail.

Since FWCST and FWBST improved power by increasing the effective proportion of causative variants, it is of interest to evaluate their performance when the effective proportion after weighting remains equal to that without weighting. This could occur when the information available does not facilitate selection of true functional variants. To remain the effective proportion equal to that without weighting, the sensitivity and specificity have to be equal (see the Discussion section for the derivation). It was clear that both FWCST and FWBST had lower power than ST (figure 7), implying that uninformative weighting schemes would cause a loss of power. The power of both tests increased with increasing sensitivity. Finally when sensitivity reached 1.0, FWBST was equivalent to ST, but FWCST was still inferior to ST. The power loss was more severe for FWBST than for FWCST in most cases.

#### Power with gene length

**Figure 8:**
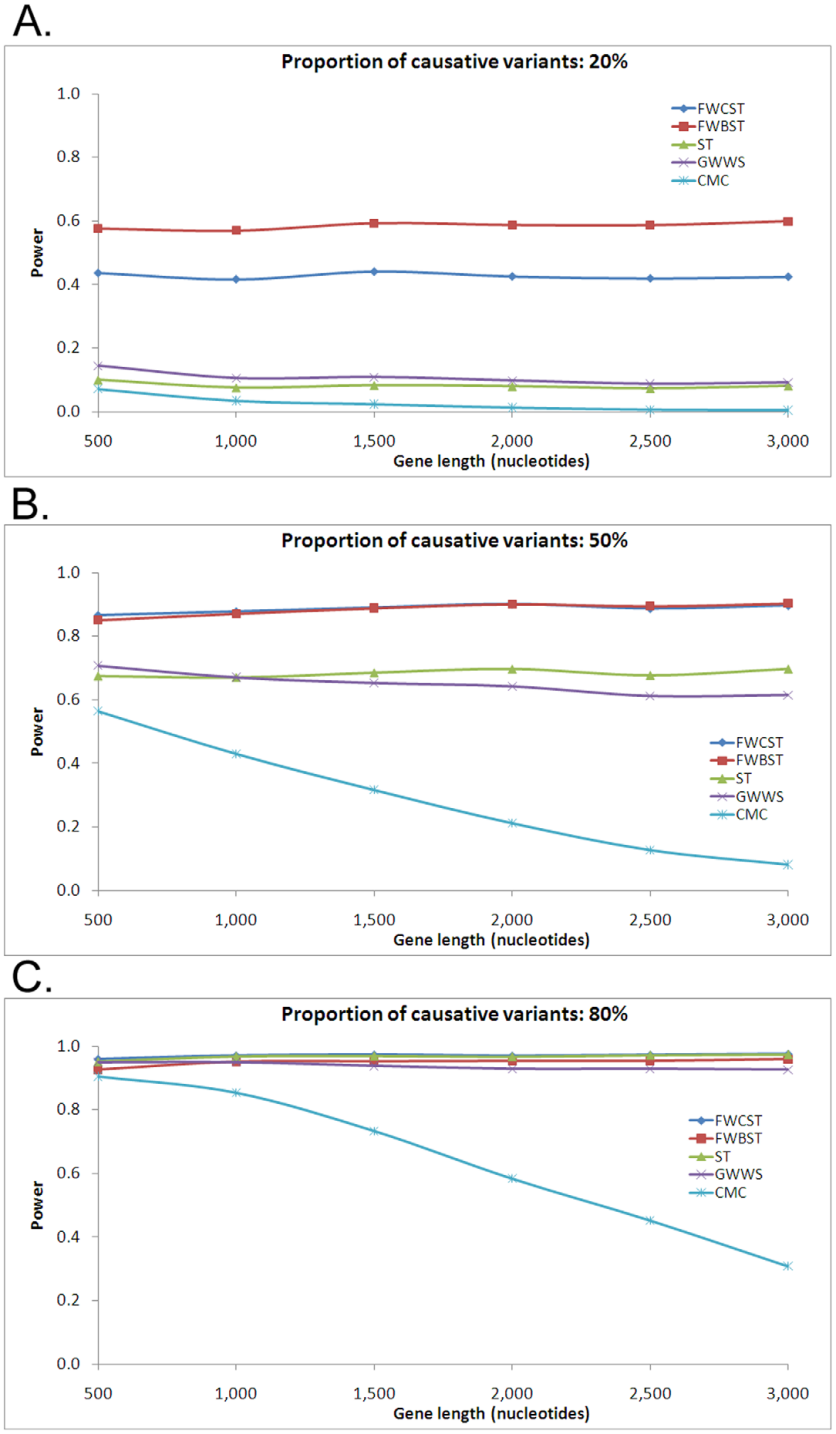
Power with gene length. Gene length varies from 500 to 3,000 nucleotides, corresponding to a protein length of 170 to 1,000 amino acids. See the legend for figure 4 for abbreviations and simulation details.

Gene length affected the performance of different tests quite differently (figure 8). For FWCST and FWBST, power increased with increasing gene length, though the magnitude of this change was minor. For the other tests, however, power decreased with increasing gene length. The power loss was much more severe for CMC than for ST and GWWS. Comparing the two functional variant weighted tests, FWBST was more powerful at low proportion (figure 8A), but had approximately equal power to that FWCST had at the other two proportions (figure 8B and 8C). At low and medium proportions, FWBST and FWCST were most powerful, followed by ST and GWWS, which had approximately equal power, and then by CMC, which had the lowest power. At the high proportion, while all other tests maintained power at high levels, the power of CMC decreased substantially with increasing gene length.

#### Power with gene effect

**Figure 9:**
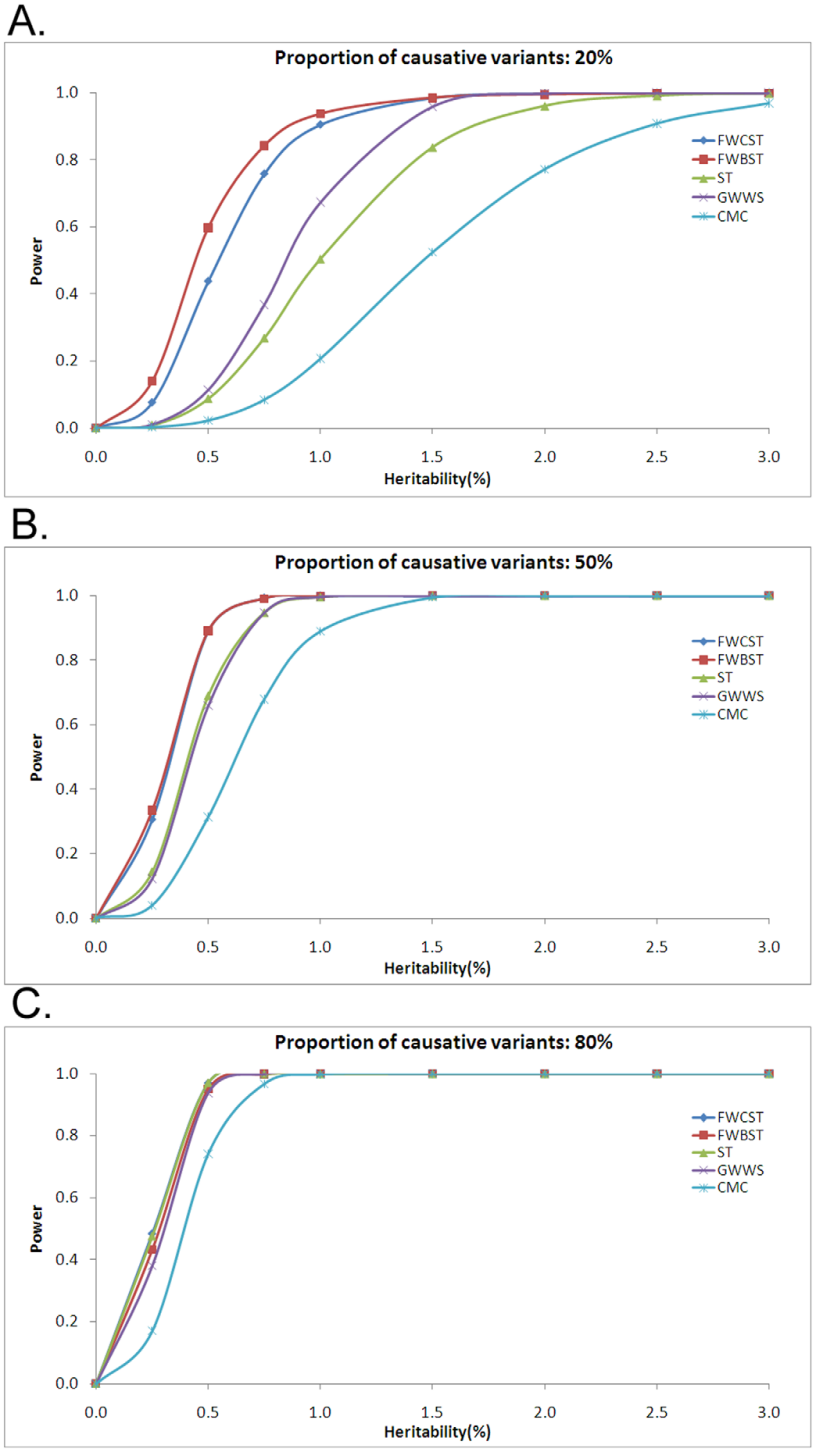
Power with gene effect. Locus heritability varies from 0% to 3%. See the legend for figure 4 for abbreviations and simulation detail.

The power of all methods increased with increasing locus heritability (figure 9). Among the tests, FWCST and FWBST were most powerful, followed by GWWS, ST, and CMC. At a low proportion of causative variants (figure 9A), FWCST, FWBST, and GWWS had the ability to detect genes with heritability of 1.5% with nearly 100% power. For ST and CMC, at a low proportion of causative variants, 100% power was achieved at heritability levels of 2.0 and 3.0%, respectively. At high proportions (figure 9C), all tests had nearly 100% power to detect genes with heritability of 1.0% or more, and similar results were obtained at medium proportions (figure 9B) with all tests except CMC, which did not achieve 100% power until heritability reached 1.5%.

#### Power with phenotyped and sequenced sample size

Since we simulated a large pool of phenotyped individuals and selected individuals with extreme phenotypes from the pool for sequencing, we investigated the effect of the number of phenotyped and sequenced individuals on power estimation. As shown in table 2, sequencing greater numbers of individuals certainly increased power for all tests, as did increasing the pool of phenotyped individuals. When sequencing 2,000 individuals from a pool of 50,000 phenotyped individuals, the power of FWCST and FWBST was 81% and 87%, respectively, for detecting a gene with 1,500 nucleotides. When the number of phenotyped individuals increased to 100,000, the power of FWCST and FWBST increased to 90% and 92%, respectively, without additional sequencing cost. Comparing the tests, the power of FWCST and FWBST was highest, followed by ST, GWWS, and CMC.

**Table 2:** Power with phenotyped and sequenced sample sizes.

Phenotyped	Sequenced	Tests				
sample size	sample size	FWCST	FWBST	ST	GWWS	CMC
25,000						
	1,000	0.29	0.41	0.05	0.06	0.01
	2,000	0.57	0.70	0.16	0.17	0.06
	4,000	0.76	0.84	0.27	0.29	0.10
50,000						
	1,000	0.44	0.59	0.09	0.11	0.02
	2,000	0.81	0.87	0.30	0.34	0.10
	4,000	0.95	0.96	0.64	0.69	0.30
100,000						
	1,000	0.60	0.72	0.15	0.20	0.04
	2,000	0.90	0.92	0.49	0.59	0.20
	4,000	0.99	0.99	0.84	0.91	0.51

The pool of phenotyped individuals with varies sizes is generated. For each pool, equal numbers of individuals with the lowest and highest phenotypes are selected for sequencing. A gene with 1,500 nucleotides is simulated. Power is estimated on 10,000 replicates at the significance level 1.0E-6.

### Application

In order to verify the results of our simulation studies, we analyzed the sequence dataset produced by Cohen et al. [34]. When applied to the Dallas sample, FWCST and FWBST had p-values of 4.03E-4 and 4.41E-4, which were higher than that of ST (2.46E-4) and GWWS (7.71E-5), but lower than that of CMC (1.17E-3) (table 3). The inferior performance of FWCST and FWBST compared to ST and GWWS was caused by the fact that of the 3 variants that were exclusive in the control group (high HDL-C), two were predicted to be “damaging protein”, resulting in a specificity rate as high as 0.7. However, this comparison probably created an advantage for un-weighted tests because another 11 variants, that were present in both case and control groups, were not included in the authors' analysis. If these variants, which were likely to be neutral, had been included, the specificity of prediction would be expected to decrease, thereby the relative performance of FWCST and FWBST compared to other tests would be improved.

**Table 3:** Analyses of the real sequence data.

Tests	p-value	
	Dallas	Canada
FWCST	4.03E-4	1.97E-4
FWBST	4.41E-4	1.46E-5
ST	2.46E-4	1.35E-3
GWWS	7.71E-5	1.81E-3
CMC	1.17E-3	2.74E-3

In the Dallas sample, three genes (*ABCA1*, *APOA1*, and *LCAT*) were sequenced in two groups, each of which contained 128 individuals. A total of 29 non-synonymous variants were identified, but only 18 variants were available for analysis. 16 of these 18 variants were exclusive to the case group, and the remaining 2 were exclusive to the control group. In the Canada sample, the same three genes were sequenced in two groups containing 155 and 108 individuals. Of the 23 variants that were available for analysis, 21 were exclusive to the case group and the remaining 2 were exclusive to the control group.

When applied to the Canada sample, both FWCST and FWBST produced lower p-values (thus likely higher power) (1.97E-4 and 1.46E-5) than those of other tests (ST: 1.35E-3; GWWS: 1.81E-3; CMC: 2.74E-3). In this sample, both variants exclusive to the control group were predicted to be neutral, resulting in a specificity rate as low as zero. Again, the comparison would be fairer if the additional 9 variants, that were present in both case and control groups, had been included for analysis.

## Discussion

In this study, we have proposed and evaluated a novel method to test the association between disease and variants in gene coding regions. Specifically, we incorporated information generated by extensive use of predicting algorithms into group-wise association tests. This was done in order to improve the power of these tests by distinguishing potential causative variants from background neutral variation. We also studied the effects of several influential factors on the performance of the proposed tests. Simulation studies showed that the use of predicting algorithms to identify potentially functional variants improves statistical power under certain conditions, especially when the proportion of functional variants is moderate. Application of the proposed tests to a real sequencing study confirmed the results of our simulation studies.

Association, referring to the correlation between phenotype and genetic variants, can be categorized into indirect and direct associations. With indirect association, the genetic variants to be examined are usually neutral bio-markers, e.g., SNPs, while the causative variants are untyped. In order for indirect association studies to be effective, there must be linkage disequilibrium (LD; i.e., nonrandom association), between these neutral bio-markers and the causative variants. Variant alleles in indirect association usually have no functional biological impact, and are often encoded as 1 or 0 to indicate the presence or absence of a particular allele. With direct association, in contrast, the variants that are examined are assumed to be functional, and not only the presence or absence of a particular allele but also its type (e.g., A, G, C, or T) is informative. This distinction has made analytical approaches for sequencing-based direct association studies different from those used for genotyping-based indirect association studies.

Sequencing genomic regions identifies a greater number of rare variants than that identified by genotyping platforms. The challenge in analyzing rare variants is attributable both to their rarity, and to the fact that a large proportion of these rare variants represent background population variation with no functional impact. While grouping strategies could enlarge mutation signals, the anticipated excess number of neutral variants within the group would decrease signal-to-noise ratios to very low levels. Therefore, it would be highly advantageous to differentiate potential causative variants from neutral ones, prior to association analyses, with the goal of increasing signal-to-noise ratios.

In gene coding regions, the functional impact of a variant can be predicted from protein structure and function, and multiple sequence alignments. Functional variants predicted by existing algorithms distribute differently between disease-affected and normal populations. For example, when applied to a disease-causing mutation database annotated in SwissProt, PolyPhen predicts that ∼82% of all mutations will result in damaged protein [19]. When applied to a control set of between-species substitutions, however, only ∼8% of mutations fall into this category [19]. Several recent sequencing studies provide additional evidence for this relationship between predicted functional variants and phenotype [30], [33], [34], [39]. For example, in the study of Cohen et al. [34], 17 of 25 non-synonymous SNPs (nsSNPs) identified in the *ABCA1* gene in case group are predicted to be “damaging protein”. Given PolyPhen's false positive rate on neutral substitutions being 0.08, the p-value for observing this data from a binomial distribution is as low as 1.30E-13. Similar analysis on nsSNPs in control group (2 of 4 being “damaging protein”) presents a much higher p-value 0.03 [27]. Therefore, predicted functional variants are highly correlated with disease phenotype, which justifies the importance and attests to the feasibility of identifying putative functional variants prior to grouped association analyses.

A variety of algorithms have been proposed to predict the functional impact of amino acid substitutions [18], [19], [20], [21], [22], [23], [24], [25], [26], [40]. Prediction could be based on multiple ortholog sequences, protein structures, and annotation of variants. In our real data analysis, we used PolyPhen [19] which uses sequence-, structure-, and annotation-based information as input. The benefits of using these prediction algorithms are dependent on the capacity to increase the effective proportion of causative variants, which is highly dependent on the sensitivity and specificity of the algorithm used. For a particular sample with *N* variants, let *f* represent the proportion of causative variants, and let 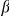 and 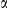 represent the sensitivity and specificity of a particular predicting algorithm. The effective numbers of causative and neutral variants after weighting are thus 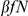 and 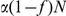, respectively, so that the effective proportion of causative variants after weighting is 
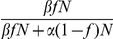
. When 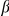
>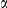, it is clear that the effective proportion will be larger than *f*, and the proposed tests tend to be more powerful. In contrast, the effective number of causative variants decreases from 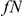 to 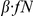, thereby reducing power. Therefore, the relative performance of weighted tests reflects the balance of these two opposite effects. When 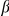 = 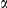, the effective proportion remains *f*, but the effective number is still reduced to 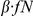. This leads to reduced power, where the power loss gets less severe with increasing 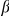, as shown by our simulations. For a particular 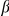, the loss of power is influenced by the diversity of weights being assigned. For the binary weighting scheme FWBST where extreme weights (1 or 0) are assigned, power loss is severe. For the continuous weighting scheme, FWCST, where intermediate continuous weights (between 0 and 1) are assigned, power loss gets less severe.

Weighting variants within a group has been proposed by Madsen & Browning [16], however, their weighting scheme is derived from the sample that is being studied. Specifically, they assigned heavier weights to rarer variants in the control population. The merit of their method relies heavily on the assumption that rarer variants have larger per-allele effects. Violation of this assumption, as observed in the simulations used in this study, would diminish the advantages of their test. In our simulations, GWWS had a greater power than ST, without weighting, only when the proportion of causative variants was low; otherwise, ST actually had a slightly greater power. When prior knowledge that is independent of the sample under investigation is available, GWWS could be inferior to the proposed functional variant weighted tests. Another assumption-related observation is that CMC is generally inferior to the other tests. This observation depends on the underlying biological mechanism for the phenotype. In CMC, individuals with one or more mutations are encoded with the same genetic score. Corresponding to the underlying mechanism of gene expression, it assumes that any single mutation will cause complete loss of protein function, which probably only occurs for non-sense mutations. In our simulations, however, mutations affect phenotype in a cumulative way such that each mutation causes only a partial loss of protein function. This critical difference between their assumption and our simulations may account for the inferior performance of CMC in this study.

Weighting variants based on prior information has also been a research focus for years in common variants based association studies [41], [42], [43], [44]. Among previous studies, Genovese et al. [42] proposed a general framework that incorporated prior information into genome-wide association studies (GWAS) to overcome the problem of multiple hypothesis testing. In brief, they assigned each SNP a weight so that SNPs with heavier weights were penalized less when using multiple testing corrections. Following their work, Roeder et al. [43] proposed formulating weights based on previous linkage traces. They divided association p-values by weights that were predefined, and applied the traditional FDR procedure to the weighted p-values. Roeder et al. [44] also proposed a weighted multiple testing procedure. Later work proposed by Chen and Witte [41] incorporated various sources of prior information, such as linkage signal, functional category, conservation, and LD, into a Bayesian hierarchical model to reflect the prior likelihood of the SNP being associated. All of these methods were shown to have the ability to improve power of detecting the association assuming that the prior information was correctly specified.

Rare and common variants aimed weightings share, in common, the idea of using prior information to improve power, however they are different in the following aspects. First, common variants are tested individually, and weighting operates on the resulting individual p-values, while rare variants are tested together and weighting operates on individual genotypes. Second, the aim of weighting for common variants is to relieve the problem of multiple hypotheses testing by punishing different tests differently through the weights they are assigned. In contrast the aim of weighting for rare variants is to improve the effective proportion of causative variants within group. Hence, largely speaking, the general goal and detailed specific strategies involved are largely different in rare and common variants aimed weightings.

In this study, we restrict our attention to non-synonymous variants in gene coding regions because these variants have direct functional implications. In principal, the weighting scheme could also be extended to variants in other genomic regions, e.g., gene introns, regulatory regions (enhancer, silencer, and etc.), splicing sites, and ultra-conserved regions, by studying ortholog sequences between and/or within species. However, caution should be taken when studying these non-coding variants, because they have no direct functional meaning in terms of affecting gene expression, and the relationship between their conservation and phenotype may not be well established. A study reported by Ahituv et al. [45] showed that after deleting four non-coding ultra-conserved elements from the mouse genome, the mice remained viable and fertile, and revealed no abnormalities for a variety of phenotypes that were studied. The effects of these conservative genomic regions in maintaining the normality of species is still being debated, and whether they can be used to direct the selection of causative variants may warrant further consideration. Focusing analyses on gene-coding regions provides a plausible trade-off between sequencing cost and discovery of informative variants, and may be an attractive study design before genome-wide sequencing study becomes widely available [14], [30], [46], [47], [48].

In conclusion, we propose that the use of predicting algorithms to distinguish causative variants from background population variation, and incorporation of this information into association tests, can improve statistical power for detecting rare variants that are associated with diseases. This conclusion is supported by both simulation studies and the application of this approach to real sequencing data. Our work may help investigators who are planning to pursue large-scale gene based sequencing studies.

## Supporting Information

Appendix S1 Demographic model.
(0.08 MB DOC)
