# Supplementary material for: Improved Detection of Rare Genetic Variants for Diseases
Source: PLoS One. 2010 Nov 8;5(11):e13857. doi: 10.1371/journal.pone.0013857 (PMC2975623; doi:10.1371/journal.pone.0013857)
Supplement: Appendix S1 — Demographic model. (0.08 MB DOC) [file pone.0013857.s001.doc]

**Appendix**

Following the work of Kryukov et al [1], let $\varphi(q,t|p,n)$ be the probability density that the allele frequency in the *t*th generation is *q* given that its frequency in the initial population (with size *n*) is *p*. For a constant population size *N*_0_, $\varphi$ is given by [1,2]

$\varphi(q,t|p,N_{0})=\sum_{i=1}^{\infty} \frac{(2i+1)(1-(1-2p)^{2})}{i(i+1)}\cdot C_{i-1}^{3/2}(1-2p)\cdot C_{i-1}^{3/2}(1-2q)\cdot e^{-\frac{i(i+1)}{4N_{0}}t}$,

where $C_{i-1}^{3/2}$ is the Gegenbauer polynomial with $\lambda=3/2$. For a exponentially growing population with $N_{t}=N_{0}\cdot e^{\gamma t}$, $\varphi$ has a solution by replacing *t* with an effective time $t^{'}=(1-e^{-\gamma t})/\gamma$, $\varphi(q,t^{'}|p,N_{0})=\varphi(q,(1-e^{-\gamma t})/\gamma|p,N_{0}).$

The probability density of allele frequency in the present population (*t* = *T*) is the sum of contributions of mutations that originated from the ancestral population before the bottleneck and that originated from the expanding population after the bottleneck

$$f(q)=4N_{1}\mu\sum_{i=1}^{2N_{b}-1} \frac{1}{i}\cdot\varphi(q,\frac{1-e^{-\gamma T}}{\gamma}|\frac{i}{2N_{b}},N_{b})+2N_{b}\mu\sum_{t=1}^{T} e^{\gamma t}\varphi(q,\frac{1-e^{-(T-t)\gamma}}{\gamma}|\frac{1}{2N_{b}e^{\gamma t}},N_{b}e^{\gamma t}),$$

where $\mu$ is the per site mutation rate. Here we set $\mu=1.8\text{E}-8$ [3].

For an observed sequence sample with *N_s_* chromosomes, the site-spectrum frequency is defined as the $\frac{N_{s}}{2}+1$ dimensional vector $\mathbf{r}=(r_{0},r_{1},...,r_{\frac{N_{s}}{2}})$, where *r_i_* is the number of sites at which the minor allele is observed *i* times. By the above demographic model, the probability that the minor allele is observed *i* ($1\leq i<\frac{N_{s}}{2}$) times is given by

$$F_{i}=\int_{0}^{1} \left(_{i}^{N_{s}}\right)(q^{i}(1-q)^{N_{s}-i}+q^{N_{s}-i}(1-q)^{i})f(q)dq,$$

and $F_{\frac{N_{s}}{2}}=\int_{0}^{1} \left(_{\frac{N_{s}}{2}}^{N_{s}}\right)(q(1-q))^{\frac{N_{s}}{2}}f(q)dq.$

The probability of observing monomorphic site is given by $F_{0}=1-\sum_{i=1}^{N_{s}/2} F_{i}.$

Assuming the independence between sites, the likelihood of the observed site-frequency spectrum data is given by

$$L(N_{1},N_{b},T,\gamma)=p(\mathbf{r}|N_{1},N_{b},T,\gamma)=\prod_{i=0}^{N_{s}/2} F_{i}^{r_{i}}.$$

Given the observed sequence data, the demographic model parameters $\theta=[N_{1},N_{b},T,\gamma]$ will be estimated by maximizing the likelihood function *L*. To estimate $\theta$, we analyze the real sequence dataset produced by the ENCODE3 project [4]. In the ENCODE3 project, ten genomic regions each comprising 100 kb sites were sequenced in many populations. Data (released on March 14^th^ 2008) were downloaded from the project ftp site (ftp://ftp.hgsc.bcm.tmc.edu/pub/data/HapMap3-ENCODE/ENCODE3/ENCODE3v1/). We use sequence data from the European population, which consists of 238 chromosomes from 119 individuals. Data from 7 genomic regions are available for analysis. Of the 700 kb genomic regions sequenced, 58.2 kb are gene-coding regions and the remaining 641.8 kb are non-coding regions. A total of 10,076 variants were reported in the raw dataset, of which 911 are in gene-coding regions. For quality control, we select variants that were successfully sequenced in all individuals, resulting in 83 gene-coding variants and 953 non-coding variants for analysis. These variants correspond approximately to 66.7 (641.8 x 953/9165) kb and 5.3 (58.2 x 83/911) kb sites sequenced in non-coding and coding regions, respectively. We use only non-coding variants to estimate the demographic model.

**References**

1. Kryukov GV, Shpunt A, Stamatoyannopoulos JA, Sunyaev SR (2009) Power of deep, all-exon resequencing for discovery of human trait genes. Proc Natl Acad Sci U S A 106: 3871-3876.

2. Williamson SH, Hernandez R, Fledel-Alon A, Zhu L, Nielsen R, et al. (2005) Simultaneous inference of selection and population growth from patterns of variation in the human genome. Proc Natl Acad Sci U S A 102: 7882-7887.

3. Kondrashov AS (2003) Direct estimates of human per nucleotide mutation rates at 20 loci causing Mendelian diseases. Hum Mutat 21: 12-27.

4. Birney E, Stamatoyannopoulos JA, Dutta A, Guigo R, Gingeras TR, et al. (2007) Identification and analysis of functional elements in 1% of the human genome by the ENCODE pilot project. Nature 447: 799-816.
